# Cardiac, Macro-, and Micro-Circulatory Abnormalities in Association With Individual Metabolic Syndrome Component: The Northern Shanghai Study

**DOI:** 10.3389/fcvm.2021.690521

**Published:** 2021-07-09

**Authors:** Fang Zhao, Rong Yang, Rusitanmujiang Maimaitiaili, Jiamin Tang, Song Zhao, Jing Xiong, Jiadela Teliewubai, Chen Chi, Jacques Blacher, Jue Li, Yawei Xu, Yan Jiang, Yi Zhang, Weiming Li

**Affiliations:** ^1^Department of Geriatrics, Shanghai Putuo People's Hospital, School of Medicine, Tongji University, Shanghai, China; ^2^Department of Cardiology, Shanghai Tenth People's Hospital, School of Medicine, Tongji University, Shanghai, China; ^3^Paris Descartes University, AP-HP, Diagnosis and Therapeutic Center, Hôtel-Dieu, Paris, France; ^4^Institute of Clinical Epidemiology and Evidence-Based Medicine, Tongji University, Shanghai, China

**Keywords:** elderly population, cardiac abnormality, macrocirculatory abnormality, microcirculatory abnormality, metabolic syndrome

## Abstract

**Objective:** This study investigated the association of metabolic syndrome (MS) and its components with cardiac, macro-, and micro-circulatory abnormalities in an elderly Chinese population.

**Methods:** This cross-sectional study was conducted using data for 1,958 participants from the Northern Shanghai Study aged over 65 years without a history of cardiovascular disease. MS was defined according to the National Cholesterol Education Program Adult Treatment Panel III in 2005 (NCEPIII 2005). Asymptomatic cardiovascular impairment parameters, including the left ventricle mass index (LVMI), peak transmitral pulsed Doppler velocity/early diastolic tissue Doppler velocity (E/Ea), carotid-femoral pulse wave velocity (cf-PWV), ankle-brachial index (ABI), carotid intima-media thickness (CIMT), arterial plaque, and urinary albumin–creatinine rate (UACR), were evaluated.

**Results:** LVMI, E/Ea, cf-PWV, and the proportion of UACR > 30 mg/g exhibited increasing trends while ABI exhibited a decreasing trend according to the number of MS components (all *p* for trend < 0.01). Logistic regression analysis revealed that MS was significantly associated with LV hypertrophy (LVH), LV diastolic dysfunction, arteriosclerosis, and microalbuminuria (all *p* < 0.001). Central obesity and high blood pressure were associated with all cardiovascular abnormalities (all *p* < 0.05), whereas elevated plasma glucose was associated with arteriosclerosis and microalbuminuria (both *p* < 0.001). In addition, high triglyceride levels were associated with microalbuminuria (*p* < 0.05).

**Conclusions:** MS is significantly associated with cardiac, macro-, and micro-circulatory abnormalities in elderly Chinese. Moreover, the presence of individual MS components may have specific prognostic significance.

## Introduction

Metabolic syndrome (MS) is characterized by a cluster of risk factors that together significantly raise the risk for cardiovascular disease (CVD), stroke, diabetes, and other health issues (1). These risk factors include central obesity (i.e., excessive accumulation of abdominal fat around the waist), hypertension, hyperglycemia, and dyslipidemia (i.e., abnormal cholesterol and/or triglyceride levels), and MS is diagnosed when three or more of the abovementioned risk factors are present (2). MS has become a significant health burden worldwide, particularly with the expansion of the aging population, because MS not only is closely related to aging (3) but also promotes precocious aging (4). In addition, the presence of MS may significantly increase the morbidity and mortality of CVD patients. For example, in a study involving 4,483 participants, MS substantially increased the cardiovascular mortality rate by 12% compared with 2.2% in those without MS (5).

Asymptomatic cardiovascular impairments, which may be reflected by the presence of cardiac, macro-, and micro-circulatory abnormalities, are considered an intermediate state between risk factors and CVD, and can develop into CVD if not managed appropriately (6). Thus, it is imperative to identify the presence of cardiac, macro-, and micro-circulatory abnormalities as early as possible so that patients with a high risk of CVD may be managed in a timely and appropriately manner. The vast majority of cardiac, macro-, and micro-circulatory abnormalities can be screened by using simple and noninvasive methods as part of risk assessment, and several clinical studies have shown the relationship between MS and one or more asymptomatic cardiovascular impairments in select populations (7–9). However, few studies have systematically examined the correlation of MS and its individual components with various asymptomatic cardiovascular impairments in the same cohort of participants (10).

Abnormal macro-circulation is commonly characterized by two forms of arterial impairments, arteriosclerosis and atherosclerosis. It is recommended that arterial stiffness should be determined noninvasively by measuring the carotid-femoral pulse wave velocity (cf-PWV) as a gold standard, and PWV > 10 m/s is defined as arteriosclerosis (11). An ankle-brachial index (ABI, the ratio of the ankle and systolic brachial pressure) <0.9 for low-limb arterial plaque and plaque in the carotid artery detected by ultrasonography are the most commonly used indexes for atherosclerosis in routine clinical practice (12). Due to the special structure of the kidney, renal micro-circulation is most susceptible to microvascular damage and the kidney is usually the site where the earliest microvascular injury occurs (13). Microalbuminuria reflects the functional status of renal micro-circulation and is commonly used to detect abnormal micro-circulation in daily clinical practice (14). In the present study, we used PWV, ABI, and the presence of carotid artery plaque as the indexes to represent abnormal macro-circulation, and microalbuminuria as the index to represent abnormal micro-circulation.

This study investigated the association between various asymptomatic cardiovascular impairments and MS as well as its components in a community-dwelling elderly Chinese population with no history of CVD. We found that MS is significantly associated with cardiac, macro-, and micro-circulatory abnormalities in elderly Chinese and that individual MS components exhibit associations with cardiac, macro-, and micro-circulatory abnormalities.

## Materials and Methods

### Ethics Statement

This study was approved by the Ethics Committee of Shanghai Tenth People's Hospital, and written informed consent was obtained from all participants.

### Study Participants

The present study is included in the Northern Shanghai Study (15). The Northern Shanghai Study (clinicaltrials.gov Identifier: NCT02368938) was a prospective study designed to establish a CV risk score based on a community dwelling Chinese elderly population, determining the profile of the associated CV risk factors and target organ damages, so as to guide the later intervention. According to *the Elderly population and cause of aging monitoring statistics of Shanghai in 2014*, the northern region has the largest population of elderly adults in Shanghai, with a total population of 1.57 million and an elderly proportion of over 19%. Thus, the northern Shanghai region, including Zhabei district and Putuo district, was selected. We used a computer-generated list of communities and randomly selected 10 communities for recruitment. The recruitment strategies included the following: (1) posting study recruitment posters in neighborhood committees and community hospitals; (2) according to the health files of the candidates, community hospitals recruited potential participants by telephone; and (3) distributing recruitment flyers directly to potential participants. When eligible individuals showed interest in participating in the study, they were sufficiently informed. Individuals who fulfilled all of the following criteria were included in the study: (1) age ≥ 65 years; (2) residents from urban communities in the northern region of Shanghai; and (3) availability for long-term follow-up. Candidates who met any of the following criteria were excluded from the study: (1) severe cardiac disease (New York Heart Association IV) or end-stage renal disease (chronic kidney disease ≥ stage 4); (2) history of stroke within 3 months; (3) a malignant tumor with a life expectancy <5 years. A total of 3,590 residents from communities in northern Shanghai were invited to participate in this study between June 2014 and 2019, and 3,363 (93.7%) participated in the initial screening process. A subsample of 2,200 participants [1,100 with MS according to the revised National Cholesterol Education Program Adult Treatment Panel III (NCEPIII) in 2005 (16) and 1,100 participants without MS], all of them without a history of CVD at baseline, were selected for the present study. This sample size was calculated based on a 1:1 exposed-to-non-exposed ratio, a cumulative incidence of 4.5–5% for cardiovascular event, an estimated hazard ratio of 2 for MS, a statistical power of 0.8, and an *a priori* defined alpha error of 0.05. Sample size was further overestimated for a possible 10% dropout rate. Finally, 968 participants with MS and 1,090 participants without MS were contacted. Of these, 935 participants with MS and 1,065 participants without MS were recruited. Of the 2,000 participants, 42 were excluded due to incomplete data. Thus, the effective sample size for our analyses was 1,958 participants (908 with MS and 1,050 without MS).

All participants were instructed to complete a standardized questionnaire to obtain information on their history of present illnesses, smoking habits, medical therapy, and family history of premature CVD. Participants who smoked at least one cigarette per day for more than 6 months were defined as smokers and those who had quit smoking for more than 12 months were defined as former smokers (17). A family history of CVD was determined using the ASSIGN criteria for premature CVD or stroke before the age of 60 years in parents or siblings (18). MS was defined according to the revised NCEPIII (2005) as having at least three of the following metabolic disorders with central obesity modified for Asian populations (16): (1) waist circumference (WC) ≥ 90 cm for men and ≥ 80 cm for women; (2) blood pressure (BP) ≥ 130/85 mmHg and/or treatment of previously diagnosed hypertension; (3) fasting plasma glucose (FPG) ≥ 5.6 mmol/L and/or previously diagnosed type 2 diabetes; (4) serum triglyceride (TG) ≥ 1.7 mmol/L and/or specific treatment for lipid abnormality; and (5) high-density lipoprotein cholesterol (HDL-c) <1.03 mmol/L for men and <1.29 mmol/L for women and/or specific treatment for lipid abnormality. The revised NCEPIII (2005) criteria were used for the statistical analyses of the association between cardiovascular impairments and MS. The study also compared the differences in this association, according to the other three MS definitions: the International Diabetes Federation (IDF), the Chinese Diabetes Society (CDS), and the Chinese Joint Committee for Developing Chinese Guidelines on Prevention and Treatment of Dyslipidemia in Adults (JCDCG). According to the IDF definition (19), MS was defined as subjects with central obesity (men: WC ≥ 90 cm; women: WC ≥ 80 cm) together with two or more of the following metabolic disorders: (1) BP ≥ 130/85 mmHg and/or treatment of previously diagnosed hypertension; (2) FPG ≥ 5.6 mmol/L and/or previously diagnosed type 2 diabetes; (3) TG ≥ 1.7 mmol/L and/or specific treatment for lipid abnormality; (4) HDL-c <1.03 mmol/L for men and <1.29 mmol/L for women and/or specific treatment for lipid abnormality. According to the CDS definition (20), MS was defined as ≥3 of the following metabolic disorders: (1) body mass index (BMI) ≥ 25 kg/m^2^; (2) BP ≥ 140/90 mmHg and/or treatment of previously diagnosed hypertension; (3) FPG ≥ 6.1 mmol/L and/or 2 h PG ≥ 7.8 mmol/L and/or treatment of previously diagnosed type 2 diabetes; (4) TG ≥ 1.7 mmol/L; and/or (5) HDL-c <0.9 mmol/L for men and <1.0 mmol/L for women. According to the 2016 JCDCG definition (21), MS was defined as ≥3 of the following metabolic disorders: (1) WC ≥ 90 cm for men and ≥85 cm for women; (2) BP ≥ 130/85 mmHg and/or treatment of previously diagnosed hypertension; (3) FPG ≥ 6.1 mmol/L and/or 2 h PG ≥ 7.8 mmol/L and/or treatment of previously diagnosed type 2 diabetes; (4) TG ≥ 1.7 mmol/L; and (5) HDL-c <1.0 mmol/L.

### Anthropometric Measurements

General physical examination was conducted on all participants, and the following data were collected: body height, WC, and BP. BMI was calculated as weight in kilograms divided by the square of height in meters. Waist and hip circumferences were measured while standing with reference to external landmarks at the narrowest section of the waist, and at the level of the largest circumference between the iliac crest to the crotch, respectively. BP was measured three times after 10 min of rest in the sitting position with a semi-automatic oscillometric device (Omron Healthcare, Kyoto, Japan) by specialized physicians based on the recommendations of the European Society of Hypertension (22), and an average of three readings was used as the final BP for data analysis.

### Biochemical Measurements

Blood and urine samples were collected after 10 h of overnight fasting to measure the FPG, total cholesterol (TC), TG, low-density lipoprotein cholesterol (LDL-c), HDL-c, alanine aminotransferase (ALT), aspartate aminotransferase (AST), serum creatinine, serum uric acid, urinary creatinine, and urinary albumin levels. Standard techniques were used to determine all of these parameters at the laboratory of Shanghai Tenth People's Hospital.

### Definitions and Measurements of Cardiovascular Impairments

#### Cardiac Assessment

Echocardiography was performed on all participants by an experienced cardiologist who was blinded to the patients' data using a MyLab 30 Gold CV machine (ESAOTE SpA, Genoa, Italy) according to the American Society of Echocardiography recommendations (23, 24). Left ventricular internal diameter at end-diastole (LVIDd) and septal (SWTd) and posterior wall thickness at end-diastole (PWTd) were measured in the parasternal long-axis view. The left ventricle mass (LVM) was calculated based on the following formula*:* LVM (g) = 0.8 × {1.04 × [(LVIDd + PWTd + SWTd)^3^ – (LVIDd)^3^]} + 0.6 (25). The LVM index (LVMI) was assessed by dividing LVM by the body surface area (BSA) (26). Left atrial parameters including the M-mode left atrial dimension (SA1) in the parasternal short-axis view and measurements of short (SA2) and long axes (LA) in the apical four-chamber view at ventricular end-systole were measured. The left atrial volume (LAV) was determined using the ellipse model formula: LAV = [π × (SA1 × SA2 × LA)/6] (27) and divided by BSA to determine the LAV index (LAVI). The peak transmitral pulsed Doppler velocity/early diastolic tissue Doppler velocity (E/Ea) was determined with continual wave Doppler in the four-chamber view and computed to evaluate the LV diastolic dysfunction.

LV hypertrophy (LVH) was defined as an LVMI ≥ 115 g/m^2^ (men) or ≥95 g/m^2^ (women). LV diastolic dysfunction was evaluated using E/Ea and other parameters of abnormal LV relaxing and filling, such as increased LVM and enlarged LAV. LV diastolic dysfunction was defined as an E/Ea ≥ 15 or 15 > E/Ea > 8 with the following: (1) LAVI > 40 ml/m^2^ and (2) LVMI > 149 g/m^2^ (men) or ≥122 g/m^2^ (women) (28, 29).

#### Macro-Circulatory Assessment

Carotid ultrasonography was performed on the common carotid arteries on both sides using a 7.5-MHz transducer to record the carotid intima-media thickness (CIMT) and the presence or absence of plaques. Ankle BPs and bilateral brachial were automatically and simultaneously determined to calculate the ABI using the VP-1000 system (Omron Healthcare, Kyoto, Japan). PWV was determined with an applanation tonometer (SphygmoCor, AtCor Medical, Sydney, Australia) by a physician who was not involved with the ultrasound examination, in accordance with the European Expert Consensus on Arterial Stiffness (11) as previously detailed (15).

Macro-circulatory abnormalities were defined as arteriosclerosis with a PWV > 10 m/s, and atherosclerosis with an ABI <0.9 or the presence of plaques (11, 12).

#### Micro-Circulatory Assessment

The urinary albumin–creatinine ratio (UACR) was determined as the ratio of urinary albumin divided by urinary creatinine. Microalbuminuria (UACR > 30 mg/g) represented a micro-circulatory abnormality.

### Statistical Analyses

The revised NCEPIII (2005) criteria were used for the statistical analyses of the association between cardiovascular impairments and MS. The normality of variables was examined with the Kolmogorov–Smirnov test. Normally distributed variables are presented as mean ± standard deviation (SD), whereas skewed variables are presented as median (interquartile range 25–75%). Categorical variables are expressed as a number with a percentage. Participants were categorized into four groups according to the number of metabolic disorders (≤ 1, 2, 3, and ≥4). One-way analysis of variance (ANOVA) was used to compare the differences in data among multiple groups, followed by the Student-Newman-Keuls (SNK) test to compare the differences in data between two groups. The Kruskal–Wallis test was used to compare skewed variables among the groups. The chi-square test was used to compare the differences in the frequency between the groups. A UACR > 30 mg/g was used as the cutoff point for trend analysis because UACR had a skewed distribution. Trends for the mean or frequency among the groups were estimated using the ANOVA test or a linear-by-linear association. The odds ratios (ORs) and 95% confidence interval (CI) were calculated to estimate the associations of various cardiovascular impairments with MS or its individual components using binary logistic regression models. All reported *p*-values were two tailed, and *p* < 0.05 were considered statistically significant. All statistical analyses were performed with the Statistical Package for Social Sciences version 13.0 (SPSS, IL, USA).

## Results

### Demographic and Baseline Clinical Characteristics of Participants

After application of the inclusion and exclusion criteria, a total of 1,958 participants (879 men and 1,079 women) were finally included in this study. The median (interquartile range) age of the participants was 68.9 (66.5–73.1) years. According to the NCEPIII (2005) criteria for MS, the prevalence of MS was 46.4% (40.8% for men and 50.9% for women, *p* < 0.001). According to the IDF criteria for MS, the prevalence of MS was 37.1% (29.9% for men and 42.9% for women, *p* < 0.001). According to the CDS criteria for MS, the prevalence of MS was 21.6% (22.8% for men and 20.6% for women, *p* = 0.244). According to the JCDCG criteria for MS, the prevalence of MS was 28.7% (30.9% for men and 26.8% for women, *p* < 0.05). According to the NCEPIII (2005) criteria for individual MS components, the number and prevalence of high BP, central obesity, elevated PG, high TG, and low HDL-c were 1,433 (73.2%), 1,097 (56.0%), 714 (36.5%), 796 (40.7%), and 694 (35.4%), respectively. The number of participants with 0, 1, 2, 3, 4, and 5 components of MS according to the NCEPIII (2005) criteria was 161 (8.2%), 379 (19.4%), 510 (26.0%), 437 (22.3%), 331 (16.9%), and 140 (7.2%), respectively. Participants were categorized into four groups according to the number of metabolic disorders: group 1, participants with ≤ 1 metabolic disorders (*n* = 540); group 2, participants with 2 metabolic disorders (*n* = 510); group 3, participants with 3 metabolic disorders (*n* = 437); and group 4, participants with ≥4 metabolic disorders (*n* = 471). The demographic and baseline clinical characteristics of the participants in each group are presented in Table 1. No differences in age, AST, TC, LDL-c, urinary creatinine, family history of premature CVD, anti-hypertensive treatment, and anti-diabetic treatment were found among the groups. However, BMI, WC, hip circumference, systolic BP (SBP), diastolic BP (DBP), ALT, serum creatinine, serum uric acid, FPG, TG, HDL-c, urinary albumin, and the proportion of male, current smokers, and lipid-lowering treatment showed differences among the four groups (all *p* < 0.05).

**Table 1:** Characteristics of the participants according to the number of metabolic disorders.

**Parameters**	**All participants (*n* = 1,958)**	**Number of metabolic disorders**				***p*-value**
		**≤1 (*n* = 540)**	**2 (*n* = 510)**	**3 (*n* = 437)**	**≥4 (*n* = 471)**	
Age (years)	68.9 (66.5–73.1)	68.6 (66.4–72.7)	69.0 (66.5–73.4)	69.1 (66.6–73.6)	69.0 (66.6–72.8)	0.550
Male, *N* (%)	879 (44.9%)	289 (53.5%)	231 (45.3%)^*^	202 (46.2%)	157 (33.3%)^*^^†^^‡^	<0.001
BMI (kg/m^2^)	24.4 ± 3.5	22.0 ± 2.6	24.5 ± 3.2^*^	25.1 ± 3.2^*^^†^	26.2 ± 3.2^*^^†^^‡^	<0.001
WC (cm)	85.9 ± 9.8	78.8 ± 7.4	86.3 ± 9.2^*^	88.1 ± 8.9^*^^†^	91.6 ± 8.6^*^^†^^‡^	<0.001
Hip circumference (cm)	96.2 ± 7.1	92.0 ± 5.7	96.8 ± 6.9^*^	97.7 ± 6.9^*^	99.1 ± 6.9^*^^†^^‡^	<0.001
SBP (mm Hg)	134.8 ± 16.7	125.7 ± 16.0	137.4 ± 16.8^*^	137.2 ±15.4^*^	140.1 ± 14.5^*^^†^^‡^	<0.001
DBP (mm Hg)	80.1 ± 9.6	76.6 ± 8.7	80.9 ± 9.7^*^	81.2 ± 9.4^*^	82.1 ± 9.5^*^	<0.001
ALT (U/L)	15.4 (12.3–20.9)	13.9 (11.1–17.8)	15.1(12.4–21.0)^*^	15.9 (12.8–20.8)^*^	17.5 (13.1–24.2)^*^^†^^‡^	<0.001
AST (U/L)	19.4 (16.8–22.8)	19.6 (16.8–22.8)	19.4 (17.2–22.8)	19.1 (16.8–22.7)	19.2 (16.4–23.2)	0.759
Serum creatinine (μmol/L)	73.6 ± 17.8	74.6 ± 16.2	73.8 ± 18.4	74.8 ± 19.4	71.3 ± 17.2^*^^‡^	<0.05
Serum uric acid (μmol/L)	328.3 ± 78.2	314.2 ± 75.5	328.1 ± 80.2^*^	331.2 ± 74.6^*^	342.1 ± 79.5^*^^†^	<0.001
FPG (mmol/L)	5.20 (4.80–5.90)	4.90 (4.70–5.30)	5.10 (4.80–5.50)^*^	5.40 (4.90–6.30)^*^^†^	6.00 (5.30–7.20)^*^^†^^‡^	<0.001
TC (mmol/L)	5.17 ± 0.98	5.13 ± 0.94	5.21 ± 0.94	5.15 ± 1.00	5.19 ± 1.06	0.556
TG (mmol/L)	1.36 (1.04–1.88)	1.08 (0.85-1.33)	1.26 (1.02–1.57)^*^	1.59 (1.17–2.07)^*^^†^	1.98 (1.54–2.62)^*^^†^^‡^	<0.001
HDL-c (mmol/L)	1.40 ± 0.36	1.59 ± 0.36	1.46 ± 0.33^*^	1.32 ± 0.31^*^^†^	1.19 ± 0.30^*^^†^^‡^	<0.001
LDL-c (mmol/L)	3.20 ± 0.86	3.15 ± 0.81	3.26 ± 0.79	3.22 ± 0.90	3.17 ± 0.93	0.160
Urinary creatinine (mmol/L)	5,884.2 ± 3,345.5	6,157.3 ± 3,467.6	5,878.7 ± 3,323.3	5,843.4 ± 3,188.6	5,612.7 ± 3,355.0	0.083
Urinary albumin (mg/L)	18.6 (8.4–27.5)	16.0 (7.2–24.7)	18.5 (8.0–26.7)^*^	19.5 (9.0–31.0)^*^	20.7 (9.5–34.5)^*^^†^	<0.001
Current smoker, *N* (%)	328 (16.8%)	109 (20.2%)	84 (16.5%)	70 (16.0%)	65 (13.8%)^*^^†^^‡^	0.003
Family history of premature CVD, *N* (%)	358 (18.3%)	91 (16.9%)	99 (19.4%)	76 (17.4%)	92 (19.5%)	0.593
Anti-hypertensive treatment, *N* (%)	814 (93.3%)	100 (95.2%)	218 (92.4%)	214 (91.5%)	282 (94.9%)	0.315
Anti-diabetic treatment, *N* (%)	268 (87.0%)	23 (88.5%)	53 (89.8%)	74 (87.1%)	118 (85.5%)	0.864
Lipid-lowering treatment, *N* (%)	239 (12.2%)	0 (0.0%)	21 (4.1%)^*^	61 (14.0%)^*^^†^	157 (33.3%)^*^^†^^‡^	<0.001

*Data were presented as mean ± standard deviation, median (interquartile range 25%−75%), or number with a percentage*.

* *vs. group 1*,

† *vs. group 2, and*

‡ *vs. group 3*.

*One-way analysis of variance, Kruskal–Wallis test, and chi-square test were used for comparisons among the four groups*.

*ALT, alanine aminotransferase; AST, aspartate aminotransferase; BMI, body mass index; CVD, cardiovascular disease; DBP, diastolic blood pressure; FPG, fasting plasma glucose; HDL-c, high-density lipoprotein cholesterol; LDL-c, low-density lipoprotein cholesterol; systolic blood pressure; TC, total cholesterol; TG, triglyceride; WC, waist circumference*.

**Figure 1:**
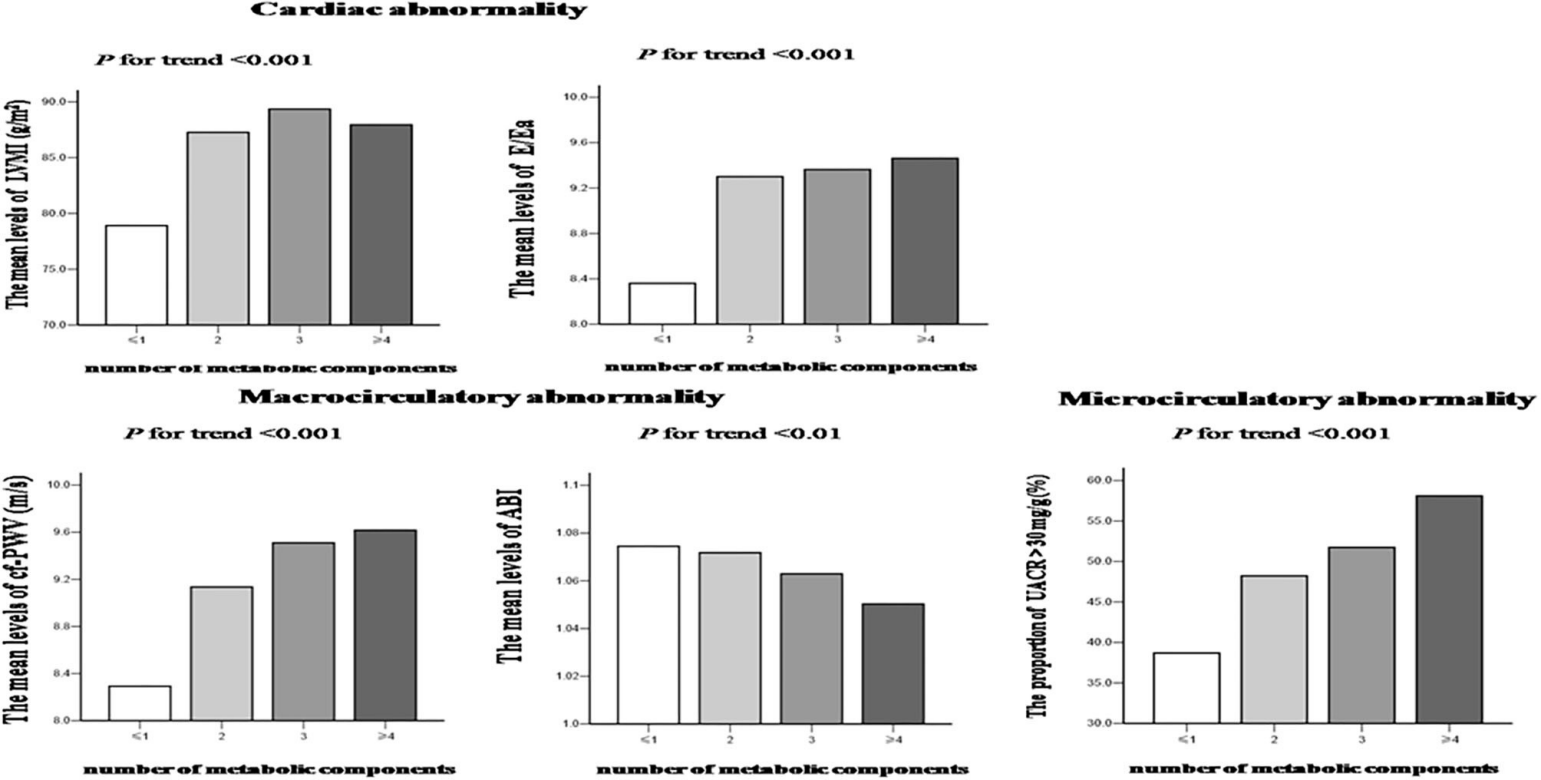
Distributions of asymptomatic cardiovascular impairments according to the number of metabolic syndrome components. A UACR > 30 mg/g was used as the cutoff point for trend analysis because UACR had a skewed distribution. Trends for the mean or frequency among the groups were estimated using the ANOVA test or a linear-by-linear association.

The characteristics of cardiovascular assessment according to the number of metabolic disorders are shown in Table 2. No differences were observed in CIMT and the frequency of plaques in the carotid artery among the groups. However, LVMI, E/Ea, cf-PWV, ABI, and UACR showed significant differences among the four groups (all *p* < 0.01). Intergroup comparisons revealed that (i) groups 2, 3, and 4 had higher LVMI, E/Ea, cf-PWV, and UACR than group 1; (ii) groups 3 and 4 had higher cf-PWV than group 2; and (iii) group 4 had higher UACR than group 2, but lower ABI than groups 1 and 2. In addition, trend analyses revealed that LVMI, E/Ea, cf-PWV, and the proportion of UACR > 30 mg/g presented an increasing trend, while ABI presented a decreasing trend according to the number of MS components (all *p* for trend <0.01; Figure 1).

**Table 2:** Characteristics of cardiovascular assessment according to the number of metabolic disorders.

**Parameters**	**All participants**	**Number of metabolic disorders**				
		**≤1 (*n* = 540)**	**2 (*n* = 510)**	**3 (*n* = 437)**	**≥4 (*n* = 471)**	***P***
**Cardiac assessment**						
LVMI (g/m^2^)	85.58 ± 27.41	78.91 ± 23.81	87.27 ± 27.74^*^	89.35 ± 27.26^*^	87.94 ± 29.73^*^	<0.001
E/Ea	9.09 ± 3.58	8.36 ± 3.17	9.30 ± 3.73^*^	9.36 ± 3.71^*^	9.46 ± 3.63^*^	<0.001
**Macrocirculatory assessment**						
cf-PWV (m/s)	9.10 ± 2.18	8.29 ± 1.86	9.14 ± 2.09^*^	9.51 ± 2.20^*^^†^	9.62 ± 2.34^*^^†^	<0.001
ABI	1.07 ± 0.12	1.07 ± 0.11	1.07 ± 0.12	1.06 ± 0.12	1.05 ± 0.12^*^^†^	0.006
C-IMT (mm)	0.67 ± 0.17	0.65 ± 0.16	0.67 ± 0.18	0.67 ± 0.18	0.68 ± 0.18	0.056
Plaque in carotid artery, *N* (%)	1,254 (64.2%)	355 (65.9%)	316 (62.5%)	279 (63.8%)	302 (64.7%)	0.708
**Microcirculatory assessment**						
UACR (mg/g)	28.96 (13.40–55.86)	22.86 (11.16–41.24)	28.81 (12.95–55.34)^*^	31.65 (14.56–62.43)^*^	37.55 (16.34–70.83)^*^^†^	<0.001

*Data were presented as mean ± standard deviation, median (interquartile range 25–75%), or number with a percentage. The characteristics of asymptomatic cardiovascular impairment according to the number of MS components were evaluated with one-way analysis of variance, Kruskal–Wallis test, and chi-square test*.

* *vs. group 1*,

† *vs. group 2*.

*ABI, ankle-brachial index; cf-PWV, carotid-femoral pulse wave velocity; C-IMT, carotid intima-media thickness; E/Ea, peak transmitral pulsed Doppler velocity/early diastolic tissue Doppler velocity; LVMI, left ventricle mass index; PP, pulse pressure; UACR, urinary albumin–creatinine rate*.

### Associations of Asymptomatic Cardiovascular Impairments With MS and Its Components

According to the definitions of cardiovascular impairment in this study, the number and prevalence of LVH, LV diastolic dysfunction, arteriosclerosis, atherosclerosis, and microalbuminuria were 404 (20.6%), 198 (10.1%), 184 (9.4%), 1,279 (65.3%), and 942 (48.1%). We next performed binary logistic regression analyses to examine the associations of various asymptomatic cardiovascular impairments with MS (Table 3). After adjustment for age (continuous variable), sex (categorical variable), smoking status (categorical variable), and family history of premature CVD (categorical variable), MS was significantly associated with LVH, LV diastolic dysfunction, arteriosclerosis, and microalbuminuria (all *p* < 0.001).

**Table 3:** Association of various asymptomatic cardiovascular impairments with MS.

**Asymptomatic cardiovascular impairment**	**Unadjusted**		Adjusted^*^	
	**OR (95% CI)**	***p*-value**	**OR (95% CI)**	***p*-value**
**Cardiac abnormality**				
LVH	1.664 (1.334–2.075)	<0.001	1.521 (1.213–1.909)	<0.001
LV diastolic dysfunction	1.904 (1.410–2.571)	<0.001	1.736 (1.279–2.356)	<0.001
**Macrocirculatory abnormality**				
Arteriosclerosis	2.278 (1.660–3.127)	<0.001	2.360 (1.699–3.279)	<0.001
Atherosclerosis	1.031 (0.855–1.243)	0.752	1.040 (0.860–1.258)	0.685
**Microcirculatory abnormality**				
Microalbuminuria	1.602 (1.338–1.918)	<0.001	1.566 (1.304–1.879)	<0.001

*The ORs and 95% CI were calculated to estimate the associations of various cardiovascular impairments with MS by using binary logistic regression models*.

* *Adjusted for age, sex, smoking status, and family history of premature CVD*.

*CI, confidence interval; CVD, cardiovascular disease; LV, left ventricle; LVH, left ventricle hypertrophy; MS, metabolic syndrome; OR, odds ratio*.

**Figure 2:**
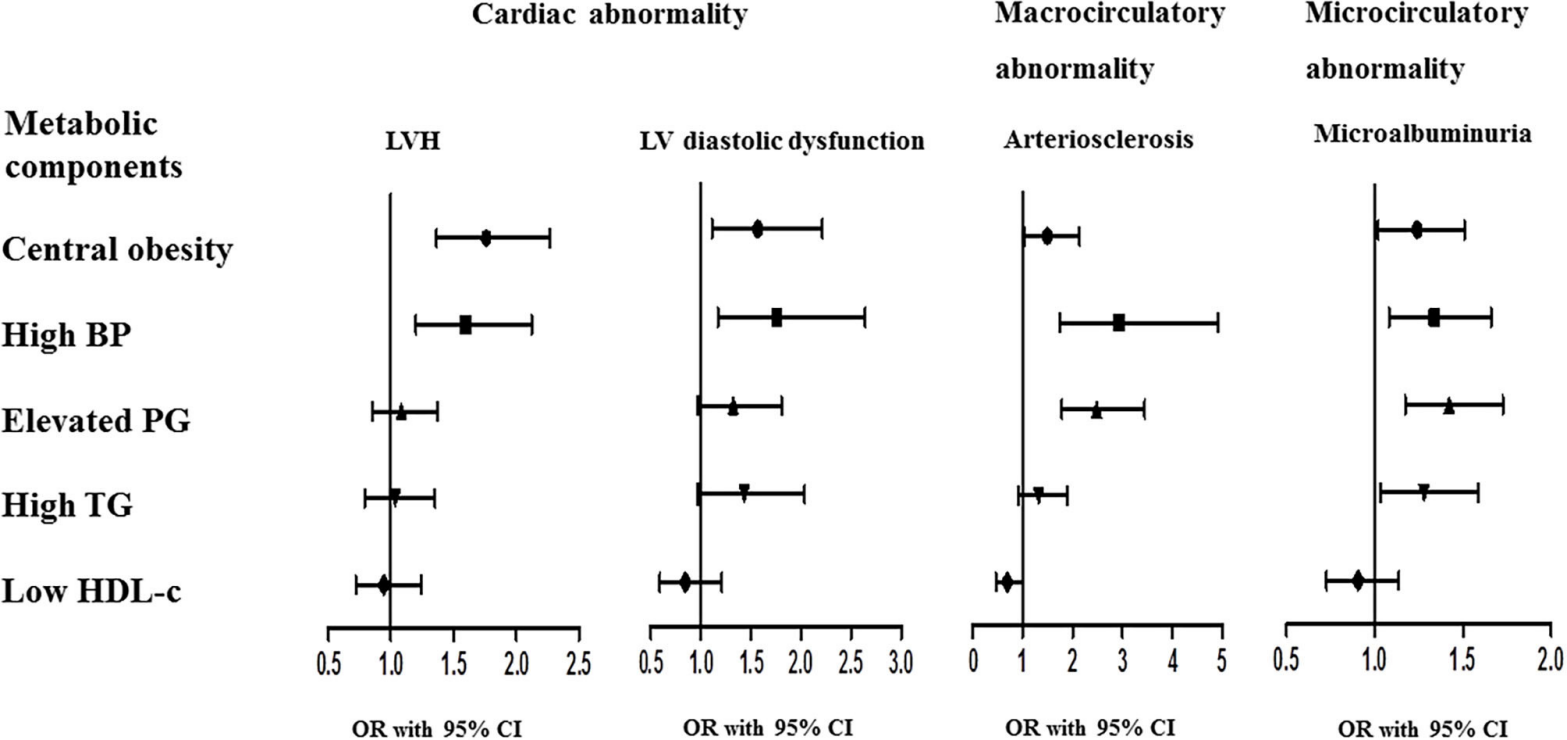
Association of asymptomatic cardiovascular impairments with individual components of metabolic syndrome. The ORs and 95% CI were calculated to estimate the associations of various cardiovascular impairments with individual components of MS using binary logistic regression models, adjusted for age, sex, smoking status, family history of premature CVD, and the other four MS components.

We also examined the associations of asymptomatic cardiovascular impairments with individual components of MS (Table 4). In model 1, MS components were separately identified as independent variables after adjustment for age (continuous variable), sex (categorical variable), serum creatinine (continuous variable), serum uric acid (continuous variable), smoking status (categorical variable), and family history of premature CVD (categorical variable). In this model, some individual MS components were associated with LVH, LV diastolic dysfunction, arteriosclerosis, and microalbuminuria. In model 2 (Figure 2), further adjustments were made for the other four MS components simultaneously (categorical variable). The results showed that (i) central obesity and high BP were associated with LVH (both *p* < 0.01); (ii) central obesity and high BP were associated with LV diastolic dysfunction (all *p* < 0.05); (iii) central obesity, high BP, and elevated PG were associated with arteriosclerosis (all *p* < 0.05); and (iv) central obesity, high BP, elevated PG, and high TG were associated with microalbuminuria (all *p* < 0.05).

**Table 4:** Association of asymptomatic cardiovascular impairments with individual components of MS.

**Variables**	**LVH**				**LV diastolic dysfunction**				**Arteriosclerosis**				**Microalbuminuria**			
	**Model 1**		**Model 2**		**Model 1**		**Model 2**		**Model 1**		**Model 2**		**Model 1**		**Model 2**	
	**OR (95% CI)**	***p*-value**	**OR (95% CI)**	***p*-value**	**OR (95% CI)**	***p*-value**	**OR (95% CI)**	***p*-value**	**OR (95% CI)**	***p*-value**	**OR (95% CI)**	***p*-value**	**OR (95% CI)**	***p*-value**	**OR (95% CI)**	***p*-value**
Central obesity	1.866 (1.451–2.400)	<0.001	1.750 (1.354–2.262)	<0.001	1.730 (1.230–2.433)	0.002	1.543 (1.091–2.182)	0.014	1.751 (1.234–2.483)	0.002	1.468 (1.025–2.102)	0.036	1.393 (1.151–1.687)	0.001	1.257 (1.032–1.532)	0.031
High BP	1.734 (1.310–2.294)	<0.001	1.577 (1.185–2.099)	0.002	1.968 (1.320–2.935)	0.001	1.744 (1.162–2.617)	0.007	3.241 (1.953–5.379)	<0.001	2.853 (1.702–4.783)	<0.001	1.490 (1.210-−1.835)	<0.001	1.340 (1.082–1.661)	0.007
Elevated PG	1.158 (0.917–1.462)	0.217	1.055 (0.830–1.340)	0.661	1.453 (1.073–1.969)	0.016	1.306 (0.957–1.783)	0.092	2.711 (1.965–3.741)	<0.001	2.561 (1.838–3.569)	<0.001	1.524 (1.261–1.842)	<0.001	1.419 (1.169–1.722)	<0.001
High TG	1.096 (0.869–1.383)	0.4401	1.023 (0.781–1.340)	0.867	1.463 (1.078–1.987)	0.015	1.414 (0.994–2.012)	0.054	1.345 (0.974–1.855)	0.072	1.289 (0.892–1.863)	0.177	1.351 (1.120–1.630)	0.002	1.293 (1.040–1.608)	0.021
Low HDL-c	1.048 (0.828–1.327)	0.697	0.949 (0.722–1.248)	0.710	1.115 (0.818–1.520)	0.490	0.853 (0.597–1.219)	0.382	0.909 (0.649–1.274)	0.579	0.685 (0.467–1.018)	0.063	1.130 (0.933–1.368)	0.212	0.913 (0.731–1.141)	0.425

*The ORs and 95% CI were calculated to estimate the associations of various cardiovascular impairments with individual components of MS by using binary logistic regression models. Model 1 was adjusted for age, sex, serum creatinine, serum uric acid, smoking status, and family history of premature CVD. Model 2 was further adjusted for the other four MS components simultaneously*.

*BP, blood pressure; CI, confidence interval; CVD, cardiovascular diseases; HDL-c, high-density lipoprotein cholesterol; LV, left ventricle; LVH, left ventricle hypertrophy; MS, metabolic syndrome; OR, odds ratio; PG, plasma glucose; TG, triglyceride*.

### Associations of Asymptomatic Cardiovascular Impairments With MS Defined by Four Criteria

We next compared the differences in this association between various asymptomatic cardiovascular impairments and MS, which was defined using the following four criteria: NCEPIII (2005), IDF, CDS, and JCDCG criteria. Logistic regression analysis with no adjustment showed that MS defined according to all four definitions was significantly associated with LVH, LV diastolic dysfunction, arteriosclerosis, and microalbuminuria (all *p* < 0.01). After adjustment for age (continuous variable), sex (categorical variable), smoking status (categorical variable), and family history of premature CVD (categorical variable), the association of MS with the aforementioned four asymptomatic cardiovascular impairments persisted (all *p* < 0.01).

## Discussion

In the present study, we examined the associations of MS and its components with various asymptomatic cardiovascular impairments in an elderly Chinese population without a history of CVD. We simultaneously assessed cardiac, macro-, and micro-circulatory abnormalities in the same population. We found that (1) these structural and functional abnormalities presented increasing or decreasing trends according to the number of metabolic disorders present; (2) MS was associated with LVH, LV diastolic dysfunction, arteriosclerosis, and microalbuminuria after adjustment for age, sex, smoking status, and family history of premature CVD; and (3) individual MS components were correlated with asymptomatic cardiovascular impairments to varying degrees.

Previously, the Strong Heart Study, a longitudinal investigation conducted in American Indian communities, analyzed the impact of NCEP III-defined MS on cardiac structure and function in 1,436 nondiabetic participants without a history of CVD, and revealed a higher prevalence of LVH in participants with MS than in those without MS (30). After adjustment for age and sex, high BP and central obesity were identified as the only two MS components associated with changes in LV mass (30). Similarly, a population survey performed in Italy showed that participants with IDF-defined MS had a higher prevalence of LVH, reduced early-to-late diastolic function, and early impairment of diastolic function compared to those without MS (31). Among the five components of MS, WC and BP were more significantly associated with LV mass (31). In line with the above findings, in the present study, we found that high BP and central obesity were strongly linked to cardiac abnormality. It is well-known that a chronic increase in LV workload in hypertensive patients can result in LVH and LV diastolic dysfunction. On the other hand, according to collective mechanistic investigations, the heart of an obese individual is chronically overloaded with an elevation in cardiac output, even in the absence of hypertension (32, 33). Therefore, it is conceivable that central obesity is significantly linked to cardiac abnormality.

PWV has been shown to reflect arterial stiffness and is a useful marker of both the severity of macro-circulatory abnormality and the prognosis of CVD in elderly individuals (34, 35). Previous studies have also shown an association between increased PWV in individuals with MS (8, 36, 37). Although studies on both animals and humans have resulted in the position of several mechanisms underlying the relationship between metabolic disorders and arterial stiffness, it was unclear which metabolic disorders were associated with PWV. In the present study, we found that high BP and central obesity were associated with PWV. It is highly likely that elevated BP accelerates arterial stiffening because it forces endothelial and arterial smooth muscle cells to be chronically exposed to increased arterial wall distensibility (38). Nevertheless, the findings regarding the association between obesity and arterial stiffness were inconsistent, and the pathophysiological processes linking abdominal adiposity to arterial stiffness were still incompletely defined (39, 40).

In the present study, we found that high BP and central obesity were also associated with microalbuminuria, which is regarded as a marker of micro-circulatory abnormality (14). Previously, Lee et al. (41) conducted a population-based cross-sectional study in Korean adults to evaluate the association between MS and microalbuminuria and found that all of the MS components were associated with an increased risk of microalbuminuria after adjustment for covariates, with high BP having the strongest and low HDL-c having the weakest association in both sexes. The mechanism underpinning the association between high BP and microalbuminuria might be related to high BP-induced renal hemodynamic changes, resulting in increased glomerular filtration, reduced tubular reabsorption of albumin, and finally structural damage to glomeruli and arterioles (42). In the present study, we also found a correlation between central obesity and microalbuminuria, which was inconsistent with the findings from a study performed in Japan (9) and the NHANES III study (43). The discrepancy in the association between central obesity and microalbuminuria between these studies was probably due to the different populations selected for the respective studies.

We showed that hyperglycemia and high BP exhibited the strongest association with PWV, which was consistent with the findings of Chen et al. in southern China (8) and a population-based study performed in Korea (41). Hyperglycemia induces pathological alterations in vascular tissues at the cellular level, which potentially accelerates the atherosclerotic process (44). We also found that hyperglycemia was the strongest determinant of microalbuminuria, which was in agreement with previous findings (9, 43).

The present study identified relatively weaker associations of high TG with microalbuminuria, whereas low HDL-c was not associated with any cardiovascular impairment. Some studies have shown that lipid-lowering treatments such as statins failed to consistently and significantly reduce the risk of major cardiovascular events (45, 46). On the other hand, HDLs have been shown to form a heterogeneous class of lipoproteins that differ in protein and lipid composition, shape, size, and density (47, 48). Therefore, there is a possibility that the association of HDL with CVD may depend on the subclasses of HDLs. Unfortunately, in the present study, we did not analyze various HDL subclasses. This might explain why low HDL-c had no correlation with any cardiovascular impairment in the present study. Our previous study showed that in an elderly community sample, non-HDL-c and TC/HDL-c had a stronger association with macro- and micro-circulatory abnormalities compared with other lipid parameters (49). However, other studies reported that HDL-c was inversely associated with the LV structure and diastolic function (50, 51). These discrepant findings merit further clarification in the future.

In our study, atherosclerosis was defined as ABI <0.9 or the presence of plaques. We found no significant association between atherosclerosis and MS, which was inconsistent with the conclusions of other studies (52, 53). Pathologically, atherosclerosis is a chronic inflammatory response to the accumulation of lipid in the artery wall and causes intimal plaques in the arteries. Although its prevalence is also age-related, the major risk factor that promotes the development of atherosclerosis is a high level of cholesterol (54). According to the definition of MS, dyslipidemia was diagnosed in participants receiving statin treatment for lipid abnormality. When participants were categorized into four groups according to the number of metabolic disorders, we found that the proportion of lipid-lowering treatment showed significant differences among the four groups [0 (0.0%), 21 (4.1%), 61 (14.0), and 157 (33.3%), *p* < 0.01], but no differences in the proportion of plaque in the carotid artery were found among the four groups. We speculated that lipid-lowering treatments such as statins significantly reduce the risk of plaques in the arteries.

**Figure 3:**
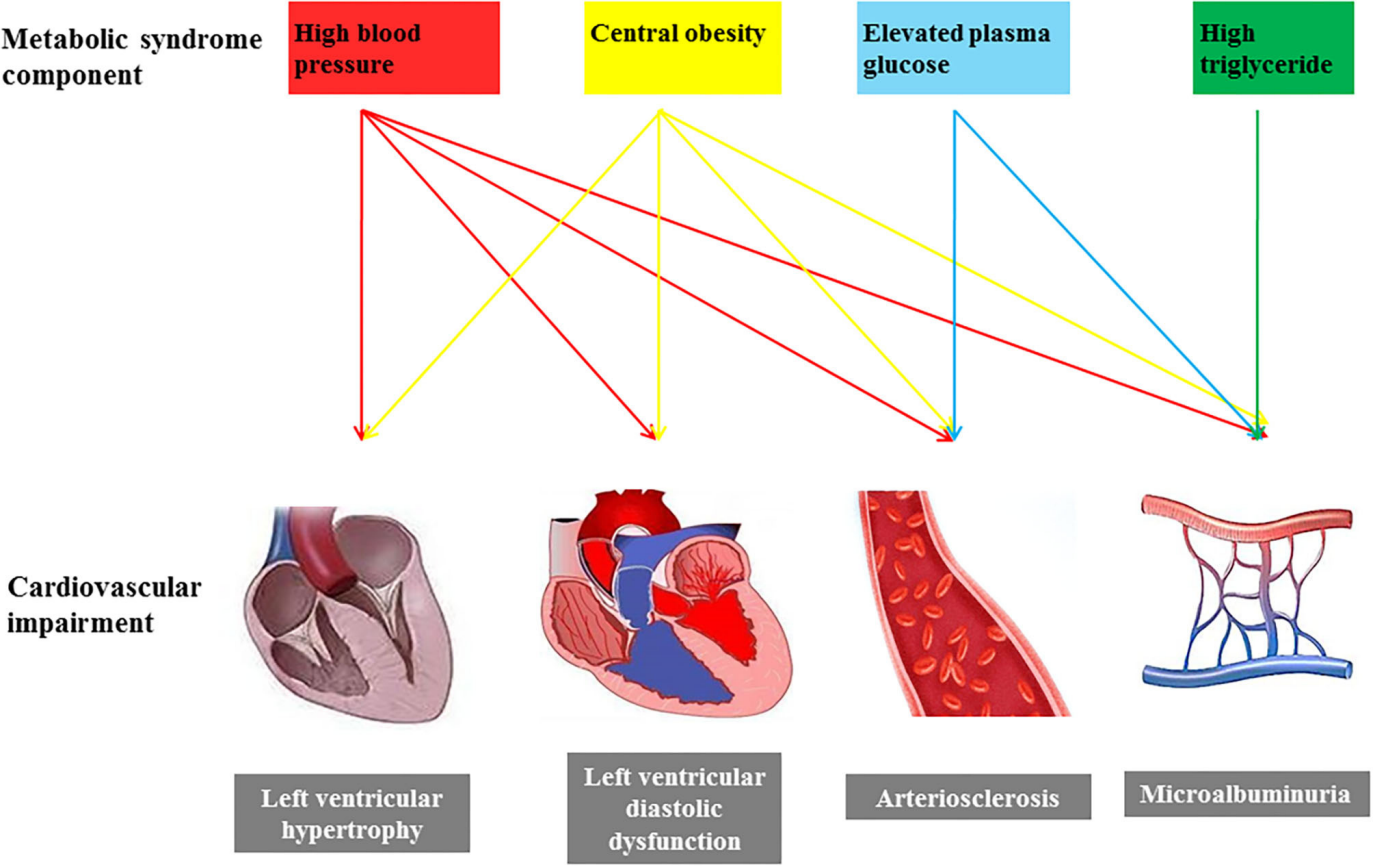
The possible effect of individual components of metabolic syndrome on asymptomatic cardiovascular impairments.

Asymptomatic cardiovascular impairment is currently regarded as an intermediate stage in the continuum of CVD and a strong determinant of total cardiovascular risk. The components of MS are related to each other and frequently appear as cluster features. A recent study in Korea showed that only high BP was significantly associated with asymptomatic cardiovascular impairment when the individual components of MS were considered (10). However, in the present study, we found that individual components of MS had different degrees of associations with asymptomatic cardiovascular impairments (Figure 3). Both high BP and central obesity were associated with all cardiovascular abnormalities, while hyperglycemia had a significant correlation with arterial stiffness and micro-circulatory abnormality. In addition, high TG was associated with micro-circulatory abnormality. We believe that identifying the association of individual MS components with cardiac, macro-, and micro- circulatory abnormalities is more important than the association of MS as a whole with cardiac, macro-, and micro-circulatory abnormalities for clinical practice. This information can help with screening and interventions for different levels of cardiovascular risk in participants with different metabolic disorders.

Although the definitions of major components of MS according to different criteria are similar, individual components of MS and their diagnostic threshold values are defined differently based on different criteria. For instance, BMI or WC is used for obesity definition and the cutoff values for FPG and HDL-c are different with different criteria. To eliminate the impacts of different definitions on MS, in the present study, we examined the associations between cardiovascular impairments and MS in our study population based on four criteria: NCEPIII 2005, IDF, CDS, and JCDCG. Interestingly, we found that MS was consistently significantly correlated with LVH, LV diastolic dysfunction, arteriosclerosis, and microalbuminuria with all four definitions we examined.

The limitations of this study should be acknowledged. First, this study was cross-sectional and hence a causal relationship between MS and cardiovascular impairments could not be identified. Second, the study population only represented a subset of the Chinese elderly population, and therefore, whether the findings obtained from this study can be generalized to other ethnicities and age groups need to be further interrogated. Third, this study did not examine the levels of 2 h post-load plasma glucose, which might affect the assessment of hyperglycemia for the definition of MS according to the CDS and JCDCG criteria. Fourth, unfortunately we did not assess retinopathy in participants using fundoscopy in the present study; thus, we were not able to evaluate the association between retinal micro-circulation and MS.

## Conclusion

In the present study, we demonstrated an association between MS and various asymptomatic cardiovascular impairments in an elderly Chinese population. Moreover, individual MS components were associated with LVH, LV diastolic dysfunction, arteriosclerosis, and microalbuminuria to varying degrees. Given the limitations of this study, prospective studies with large cohorts are warranted in the future to further clarify the role of these metabolic disorders in the development of asymptomatic cardiovascular impairments.

## Data Availability Statement

The raw data supporting the conclusions of this article will be made available by the authors, without undue reservation.

## Ethics Statement

The studies involving human participants were reviewed and approved by the ethics committee of Shanghai Tenth People's Hospital. The patients/participants provided their written informed consent to participate in this study.

## Author Contributions

YZ and WL formulated the methods and designed the protocol. FZ and RY drafted the manuscript. RY and YZ contributed toward data analysis. YZ, WL, and FZ revised the article. All authors read the article and agree to be accountable for all aspects of the work.

## Conflict of Interest

The authors declare that the research was conducted in the absence of any commercial or financial relationships that could be construed as a potential conflict of interest.
